# Conditioned medium from primary cytotrophoblasts, primary placenta-derived mesenchymal stem cells, or sub-cultured placental tissue promoted HUVEC angiogenesis in vitro

**DOI:** 10.1186/s13287-021-02192-1

**Published:** 2021-02-17

**Authors:** Haiying Ma, Shenglu Jiang, Lili Du, Jinfang Liu, Xiaoyan Xu, Xiaomei Lu, Ling Ma, Hua Zhu, Jun Wei, Yanqiu Yu

**Affiliations:** 1 Department of Pathophysiology, College of Basic Medical Science, China Medical University, No.77, Puhe Road, Shenyang North New Area, Shenyang, 110122 Liaoning Province China; 2 Department of Pathophysiology, Zhangjiakou University, No.P19, Pingmen Street, Qiaoxi District, Zhangjiakou, 075000 Hebei Province China; 3 Department of Basic Medical Sciences, Basic Medical College, Shan Xi University of Traditional Chinese Medicine, No. 89, Section 1, Jinci Road, Taiyuan, 030024 Shanxi Province China; 4 Department of Obstetrics and Gynecology, Shengjing Hospital of China Medical University, No.36 Sanhao Street, Heping District, Shenyang, 110004 Liaoning Province China; 5 Shenyang Engineering Technology R&D Center of Cell Therapy CO.LTD, No. 400-8, Zhihui 2nd Street, Hunnan District, Shenyang, 110169 Liaoning Province China

**Keywords:** Conditioned medium, Primary cytotrophoblasts, Primary human placenta-derived mesenchymal stem cells, Placental tissue, HUVECs, Angiogenesis

## Abstract

**Background:**

As a large capillary network, the human placenta plays an important role throughout pregnancy. Placental vascular development is complex and delicate and involves many types of placental cells, such as trophoblasts, and mesenchymal stem cells. There has been no systematic, comparative study on the roles of these two groups of placental cells and the whole placental tissue in the placental angiogenesis. In this study, primary cytotrophoblasts (CTBs) from early pregnancy and primary human placenta-derived mesenchymal stem cells (hPDMSCs) from different stages of pregnancy were selected as the cell research objects, and full-term placental tissue was selected as the tissue research object to detect the effects of their conditioned medium (CM) on human umbilical vein endothelial cell (HUVEC) angiogenesis.

**Methods:**

We successfully isolated primary hPDMSCs and CTBs, collected CM from these placental cells and sub-cultured placental tissue, and then evaluated the effects of the CM on a series of angiogenic processes in HUVECs in vitro. Furthermore, we measured the levels of angiogenic factors in the CM of placental cells or tissue by an angiogenesis antibody array.

**Results:**

The results showed that not only placental cells but also sub-cultured placental tissue, to some extent, promoted HUVEC angiogenesis in vitro by promoting proliferation, adhesion, migration, invasion, and tube formation. We also found that primary placental cells in early pregnancy, whether CTBs or hPDMSCs, played more significant roles than those in full-term pregnancy. Placental cell-derived CM collected at 24 h or 48 h had the best effect, and sub-cultured placental tissue-derived CM collected at 7 days had the best effect among all the different time points. The semiquantitative angiogenesis antibody array showed that 18 of the 43 angiogenic factors had obvious spots in placental cell-derived CM or sub-cultured placental tissue-derived CM, and the levels of 5 factors (including CXCL-5, GRO, IL-6, IL-8, and MCP-1) were the highest in sub-cultured placental tissue-derived CM.

**Conclusions:**

CM obtained from placental cells (primary CTBs or hPDMSCs) or sub-cultured placental tissue contained proangiogenic factors and promoted HUVEC angiogenesis in vitro. Therefore, our research is helpful to better understand placental angiogenesis regulation and provides theoretical support for the clinical application of placental components, especially sub-cultured placental tissue-derived CM, in vascular tissue engineering and clinical treatments.

## Background

As a vascularized organ, the human placenta plays an important role in the transport of oxygen, nutrients, and waste products between the mother and fetus throughout pregnancy [1]. From egg fertilization until the end of the pregnancy, the placenta develops into a capillary network of more than 550 km of vessels and 15 m^2^ in area [2, 3]. Placental vascular development is a complex and delicate regulatory process that is not only controlled by the synergistic effect of various signaling molecules, such as proangiogenic factors and antiangiogenic factors but also influenced by many placental cell types [2–7].

It is well known that placental villi consist of different cell types: (1) trophoblasts (syncytiotrophoblasts and cytotrophoblasts), (2) mesenchymal cells (mesenchymal stem cells, fibroblasts, mesenchymal-derived macrophages—Hoffbauer cells), and (3) fetal vascular cells (vascular smooth muscle cells, vascular pericytes, and endothelial cells) [4]. These cells are so close together that they even share a basement membrane in third-trimester pregnancy [8]. Therefore, proper development of the placental vasculature depends on the autocrine and paracrine signals of vascular cells and other types of placental cells [9].

In addition to differentiating into endothelial progenitor cells to form placental blood vessels, human placenta-derived mesenchymal stem cells (hPDMSCs) can also produce various soluble growth factors and cytokines to regulate the placental vasculature [9–15]. For example, some researchers have found that hPDMSCs can secrete many angiogenic factors to regulate trophoblast migration or endothelial cell angiogenesis in the context of placental angiogenesis [10, 16].

Villous trophoblasts form the traditional placental barrier between the mother and fetus and may be the main sources of these proangiogenic and antiangiogenic factors. Some researchers believe that trophoblasts can secrete angiogenic factors to recruit and maintain angiogenic cells. Trophoblasts may regulate placental vascularization through the interaction between angiogenic factors expressed by trophoblasts and their receptors on endothelial cells [17, 18].

Our research group has studied hPDMSCs for more than 10 years [19–24]. To study the regulation of placental angiogenesis, the two most plentiful types of placental cells, cytotrophoblasts (CTBs) and mesenchymal stem cells, were successfully isolated. Primary CTBs from early pregnancy and primary hPDMSCs from different stages of pregnancy were used as the cell research objects, sub-cultured full-term placental tissue was used as the tissue research object, and human umbilical vein endothelial cells (HUVECs) were used as the cell model for in vitro angiogenesis studies. Conditioned medium (CM) of placental cells and sub-cultured placental tissue was collected to determine their effect on a series of angiogenic processes in HUVECs in vitro, including proliferation, adhesion, migration, invasion, and tube formation. The angiogenic factors in the CM of placental cells and tissue were measured by an angiogenesis factor antibody array. Our results elucidate the mechanism of placental angiogenesis and provide theoretical support for the clinical application of placental components in vascular regeneration.

## Methods

### Placental sample collection

The placental tissues of first-trimester pregnancy (6–12 weeks) and second-trimester pregnancy (13–34 weeks) were obtained from the legal abortion and those of full-term pregnancy (36–40 weeks) were obtained by the cesarean section. All placental specimens were collected from the Department of Obstetrics and Gynecology of Shengjing Hospital of China Medical University (Shenyang, China). This study was approved by the Ethics Committee of China Medical University. All participants were healthy women between the ages of 25 and 40 years and had no history of infection, underlying disease, or obstetric complications.

The blood-free villi of early pregnancy and the avascular villi between the maternal side and fetal side in the second-trimester and full-term pregnancy were selected. Each placental sample was collected immediately after miscarriage or delivery and then placed in cold phosphate-buffered saline (PBS).

### Cell lines

The HTR-8/SVneo cell line was transfected with simian virus 40 large T antigen (SV40) using normal human first-trimester extravillous cytotrophoblasts (evCTBs) [25, 26]. HUVECs derived from the umbilical vein endothelium were used as a laboratory model for the study of endothelial cell functions, such as angiogenesis. The HTR-8/SVneo cell line (Chinese Academy of Sciences, China) and HUVECs cell line (Chi Scientific, China) were cultured in high-glucose Dulbecco’s modified Eagle’s medium (DMEM, Invitrogen, USA) supplemented with 10% fetal bovine serum (FBS, Gibco, USA), 2 mM l-glutamine (Invitrogen, USA), and 1% penicillin/streptomycin (Gibco, USA) in a 5% CO_2_ incubator at 37 °C (standard culture conditions). The culture medium was replaced every 3–5 days. After the cells reached 80% confluence, the cells were harvested with 0.25% trypsin (Gibco, USA) and stored in a − 80 °C freezer for subsequent experiments.

### Primary hPDMSCs and primary CTB isolation, culture, and identification

#### Primary hPDMSC isolation and culture

hPDMSCs were obtained according to our previous study protocol [22, 23]. The placental villi tissue from the different pregnancy periods was dissected into small pieces and extensively rinsed in PBS with 5% penicillin/streptomycin to remove the blood. After being mechanically minced again, the minced placental tissue was laid uniformly in 10-cm^2^ Petri dishes until semidry and cultured in DMEM supplemented with 10% FBS, 2% penicillin/streptomycin, and 2 mM l-glutamine under standard culture conditions. The next day, the supernatant was discarded, the cells were washed with PBS three times, and a new medium was added. After 3–7 days, the cells crawled out of the tissue mass. The culture medium was replaced every 5 days. After 15–21 days, the placental tissue fragments were removed, and hPDMSCs were digested with 0.25% trypsin and passaged. While the cells reached 80% confluence, they were passaged at a ratio of 1:2. hPDMSCs at passages 3–5 were stored a − 80 °C freezer and used subsequent experiments.

#### Primary hPDMSC identification

Primary hPDMSCs at passage 3 (P3) were used to measure cell surface marker expression and differentiation capacity.

#### Immunophenotype analysis of primary hPDMSCs

hPDMSCs were harvested with trypsin, washed twice with PBS, centrifuged at 1000 rpm for 10 min, and resuspended in 1 ml of PBS at a final concentration of 5 × 10^5^/ml. Then, the harvested cells were incubated with the following phycoerythrin (PE)-, fluorescein isothiocyanate (FITC)-, or PerCP-conjugated antibodies for 30 min at 4 °C: anti-CD34-PE, anti-CD45-FITC, anti-CD73-PE, anti-CD90-PE, and anti-CD105-PerCP (all from B&D, USA). After being incubated, the cells were washed, centrifuged, suspended in PBS, and analyzed by flow cytometry.

### Differentiation of primary hPDMSCs

#### Endothelial cell differentiation

For endothelial cell differentiation, primary hPDMSCs were cultured in endothelial differentiation medium. The medium was prepared with DMEM with 0.1 mM β-mercaptoethanol (Sigma, USA), 50 ng/ml recombinant human vascular endothelial growth factor (rh-VEGF, Sigma, USA), 10 ng/ml basic fibroblast growth factor (rh-bFGF, Sigma, USA), and 5% FBS. The differentiation medium was replaced every 3–5 days. On the 8th–10th day, the differentiated cells were fixed with 4% paraformaldehyde, and immunocytochemical analysis was performed with mouse anti-human von Willebrand factor (vWF, Maxim, China) according to the manufacturer’s instructions [22].

#### Osteogenic differentiation

To detect the osteogenic differentiation ability of hPDMSCs, hPDMSCs were cultured in osteogenic differentiation medium and identified by Alizarin red according to the manufacturer’s instructions for the human umbilical cord mesenchymal stem cell osteogenic differentiation kits (Chem-bio, China). Ascorbic acid, β-glycerophosphate, dexamethasone, penicillin/streptomycin, and FBS were added to the basal medium to form the complete osteogenic induction medium. When the hPDMSCs were more than 60% confluent, they were cultured in complete osteogenic induction medium. The induction medium was replaced every 3 days for 2–4 weeks. After induction, the cells were fixed with 4% paraformaldehyde for 20 min, stained with Alizarin red solution for 15 min, and imaged.

#### Adipogenic differentiation

Similar to osteogenic differentiation, adipogenic differentiation and oil red O detection were performed using an adipogenic differentiation induction kit according to the manufacturer’s instructions. First, dexamethasone, rosiglitazone, 3-isobutyl-1-methylxanthine, insulin, penicillin/streptomycin, and FBS were added into the basal medium to form complete adipogenic differentiation induction medium A. Adipogenic differentiation maintenance medium B was formed by adding insulin, penicillin/streptomycin, and FBS to the basal medium. When hPDMSCs were 80% confluent, the cells were cultured with complete adipogenic induction medium A for 72 h, and then the medium was replaced with maintenance adipogenic induction medium B and further incubated for 24 h, which was one cycle. Three to five cycles were repeated. When obvious lipid droplets appeared in the cell, the cells were cultured only in adipogenic differentiation maintenance medium B, and new medium B was replaced every 2 days. When the lipid droplets were large enough, the culture ended. Then, the induced cells were fixed with paraformaldehyde, stained with oil red O, and imaged.

### Primary first-trimester CTB isolation, culture, and identification

#### Primary CTB isolation and culture

Primary single CTBs were isolated from human first-trimester villi as described previously with some modifications [27, 28]. First, following the same steps as those of primary hPDMSC isolation, the placental villi were minced into small pieces and rinsed extensively with PBS. Then, the digestion mixture containing 0.25% trypsin and 0.1 mg/ml DNAse I (Biodee, China) was added and incubated in a 37 °C shaking water bath for 30 min. The digested suspension was filtered through a nylon mesh (200 μm) and terminated with FBS. The remaining tissue was digested with the digestion mixture containing 1 mg/ml collagen I (Invitrogen, USA) and 0.1 mg/ml DNAse I in a 37 °C shaking water bath for 20 min. The digested suspension was filtered, and digestion was terminated again. The whole digestion procedure was repeated at least 3 times. Then, the whole-cell suspension was filtered through a nylon mesh (100 μm) and centrifuged at 1000 rpm for 10 min. The cell pellet was resuspended in 3 ml DMEM with 10% FBS, gently layered on the top of a preformed noncontinuous Percoll gradient (Phamaiva, USA) (75–25%), and centrifuged at 3000 rpm for 30 min. Single cells were collected between the 45 and 35% Percoll aliquots, resuspended, and counted. The freshly isolated cell suspension was seeded at a concentration of 1 × 10^8^ cells/dish into 10-cm^2^ Petri dishes to collect CM or analyze by flow cytometry (or at a concentration of 1 × 10^5^ cells/dish into 3-cm^2^ Petri dishes for identification). After being cultured for 1 h, the cell suspension was transferred to new Petri dishes and incubated overnight. The next day, the nonadherent cells were removed, and a new medium was added. Once the cells in the 10-cm^2^ Petri dishes were more than 90% confluent, the medium was replaced with serum-free DMEM, and the primary CTBs-CM was collected at different time points. The isolated cells in the 3-cm^2^ Petri dishes were passaged for identification.

#### Primary first-trimester CTB identification

Immunofluorescence staining was performed as previously described [16, 29]. Briefly, human first-trimester placental tissue sections, primary CTBs, or HTR-8 cells were fixed, blocked, and incubated at 4 °C overnight with rabbit anti-cytokeratin 7 (CK7, Abcam, USA) and mouse anti-vimentin (Vim, Abcam, USA) to identify trophoblasts. The nuclei were stained with 4′, 6-diamidino-2-phenylindole (DAPI; Sigma-Aldrich, USA). The placental sections, primary CTBs, or HTR-8 cells were observed under the fluorescence microscope (Leica, Germany), and pictures were taken. At the same time, the harvested primary CTBs were incubated with anti-CK7 and anti-Vimentin and analyzed by flow cytometry.

### Conditioned medium preparation and experiments

#### Placental cell-derived conditioned medium

hPDMSCs at passages 3 to 5 and isolated primary CTBs cultured for 1–2 days were used to collect the conditioned medium. To harvest the conditioned medium, the cells were cultured with 10% FBS in DMEM until the cells were greater than 90% confluent and washed with PBS to remove detached cells. Then, the medium was replaced with serum-free DMEM. At different time points (6, 12, 24, 48, and 72 h), the cell culture supernatant was collected and centrifuged at 3500 rpm for 20 min to remove detached cells and cellular debris. Then, the placental cell CM was filtered (0.22 μm), adjusted with serum-free medium to 10 ml/5 × 10^7^ cells, and frozen at − 80 °C for future experiments.

#### Sub-cultured placental tissue-derived conditioned medium

The nonvascular placental stromal tissue between the maternal surface and fetal surface was selected for the collection of sub-cultured placental tissue conditioned medium. These placental samples were mechanically minced into small pieces and washed with PBS supplemented with 5% penicillin/streptomycin. Then, the wet placental villi tissue (1 cm^3^/dish) was laid uniformly in a 10-cm^2^ Petri dish until the tissue was semidry, and serum-free DMEM was added. The next day, the supernatant was discarded, the tissue was washed, and the medium was replaced with 10 ml of serum-free DMEM. At different time points (1, 3, 5, 7, 10, and 14 days), the supernatant of the cultured placental tissue was collected, centrifuged, filtered, and then adjusted with serum-free medium to 10 ml/dish. Finally, the sub-cultured placental tissue-derived CM was stored at − 80 °C for further experiments.

### Experimental groups

All in vitro HUVEC experiments were performed with conditioned medium from placental cells or sub-cultured full-term placental tissue that was collected at different time points.

Placental cell types included primary cytotrophoblasts from early pregnancy placenta (early-CTBs), human mesenchymal stem cells from early pregnancy placenta (early-hPDMSCs), human mesenchymal stem cells from middle pregnancy placenta (middle-hPDMSCs), and human mesenchymal stem cells from full-term placenta (term-hPDMSCs).

The different time points included 6, 12, 24, 48, and 72 h for placental cells, and 1, 3, 5, 7, 10, and 14 days for sub-cultured placental tissue.

### HUVEC proliferation assay

First, HUVECs were seeded into 24-well plates at a concentration of 1.0 × 10^4^ cells/well. The cells were cultured overnight in a standard culture medium supplemented with 10% FBS, the medium was discarded, and 1 ml of the conditioned medium from the different experimental groups supplemented with 5% FBS (only DMEM was used as the control) was added. At different time points (placental cell-derived conditioned medium groups: 1, 2, 3, to 6 days; sub-cultured placental tissue-derived conditioned medium groups: 12, 24, 36, and 48 h), the cells were harvested and counted. The proliferation ability of HUVECs was measured by counting the number of cells using a Countstar automated cell counter (ALIT Life Science, USA).

### HUVEC adhesion assay

To assess the adhesion ability of HUVECs, a cell adhesion assay was performed with slight modifications according to WOOD [30]. Briefly, the 96-well plates were coated with conditioned medium (50 μl/well) overnight in a 5% CO_2_ incubator at 37 °C. The next day, the CM was removed, and the 96-well plates were air-dried in the biosafety cabinet. Then, HUVECs (0.5 × 10^3^ cells/well) were suspended in 100 μl of conditioned medium and seeded into the corresponding CM-coated 96-well plates. After being incubated for 2 h, the cells were washed to remove nonadherent cells. Adherent cells were fixed with 4% paraformaldehyde and stained with 0.1% gentian violet. Three to five visual fields per well were randomly selected to take pictures and count cells.

### HUVEC wound healing assay

The horizontal migration ability of HUVECs was evaluated by using a wound healing assay. The cells were seeded into 24-well plates and grown to confluence. The confluent cells were scratched with a pipette tip, washed three times with PBS to remove the loose cells, and photographed. After being incubated for 18 h in different CM with 5% FBS, the cells were fixed, and pictures were taken in 3 random fields. The cell-free area between the confluent cells was measured by ImageJ (NIH, USA) and calculated by the following formula:

The cell-free clear distance (vertical scratch in the picture) = the clear area/height of the picture; the cell-free clear distance (horizontal scratch in the picture) = the clear area/width of the picture.

The migration distance = the cell-free clear distance at 0 h–the cell-free clear distance at 18 h.

The ratio of the migration distance between the experimental group and the control group was calculated as the relative ratio of the migration distance, which represents cell migration ability [31, 32].

### HUVEC transwell migration assay

HUVEC vertical migration was assessed by using a transwell migration assay, which was performed with a 24-well transwell insert (8 μm pore size, Corning Costar, USA). Next, 250 μl of different conditioned media containing 20% FBS was added to the lower chamber, and 100 μl of HUVEC suspension containing 3% FBS was seeded into the upper chamber at a density of 2 × 10^4^ cells/well. After being incubated for 18 h, the nonmigratory cells on the upper surface of the membrane were gently removed with cotton swabs. The migrated cells on the lower surface of the membrane were fixed with 4% paraformaldehyde and stained with 0.1% gentian violet. Three to five areas were randomly selected to take pictures and count [33, 34].

### HUVEC transwell invasion assay

The transwell invasion assay was carried out with Matrigel invasion chambers with 8.0 μm PET membranes (Corning Biocoat, USA). HUVEC suspension (100 μl, 8 × 10^4^ cells/well) supplemented with 3% FBS were seeded into the upper Matrigel-coated chamber, and 250 μl of different conditioned media supplemented with 20% FBS was added into the lower chamber. Then, the following steps were the same as those in the transwell migration assay. After being incubated for 18 h, the noninvasive cells were removed, and the invasive cells were fixed, stained, and photographed.

### In vitro Matrigel tube formation assay

The angiogenic ability of HUVECs in vitro was detected by using the Matrigel tube formation assay. Briefly, Matrigel (50 μl/well, BD, USA) was added to precooled 96-well plates and polymerized at 37 °C for 2 h to form a thin gel layer. DMEM supplemented with VEGF-A was used as a positive control, and DMEM without any additive was used as a negative control. HUVECs (3 × 10^4^ cells/well) were suspended in a conditioned medium supplemented with 2% FBS and seeded onto 96-well plates containing Matrigel. After being incubated for 15 h, the capillary-like structures were observed under a light microscope, and pictures of 3–5 visual fields/well were taken. The total length of the tubular structure was analyzed by using the Angiogenesis Analyzer plug-in for ImageJ software (NIH, USA) [35, 36].

### Angiogenesis antibody array analysis of placental cell- or sub-cultured placental tissue-derived conditioned medium

Angiogenic cytokines/proteins in the conditioned medium were analyzed with a human angiogenesis antibody array C series 1000 kit (RayBiotech, USA). This array consists of two membranes containing 43 factors related to angiogenesis, each of which is repeated twice. The cytokine array protocol was carried out according to the manufacturer’s instructions. Briefly, the array membrane was blocked with 5% BSA for 30 min and incubated in 1 ml of the conditioned medium at 4 °C overnight. After being washed thoroughly to remove unbound substances, the membrane was incubated with a cocktail of biotin-labeled cytokine antibodies at room temperature for 2 h. Then, the membrane was incubated with HRP-streptavidin at room temperature for 1 h. Finally, the chemiluminescent signal of each factor on the array was acquired by ChemiDoc XRS (Bio-Rad, USA), and the value of integrated optical density (IOD) was measured with ImageJ software. The IOD value of each spot was corrected by subtracting the background value and was standardized with the IOD value of the positive control spots on the same membrane to obtain the relative level of each factor [12, 37, 38].

### Statistical analysis

All experiments were repeated three times. All results are expressed as the mean ± SD. Statistical analysis was performed with Prism 7 software (GraphPad, USA). One-way ANOVA was performed to compare the differences within groups, and two-way ANOVA was used to assess the difference between groups. Statistical significance was considered as *p* < 0.05.

## Results

### Characterization of primary placental mesenchymal stem cells and cytotrophoblasts

**Fig. 1:**
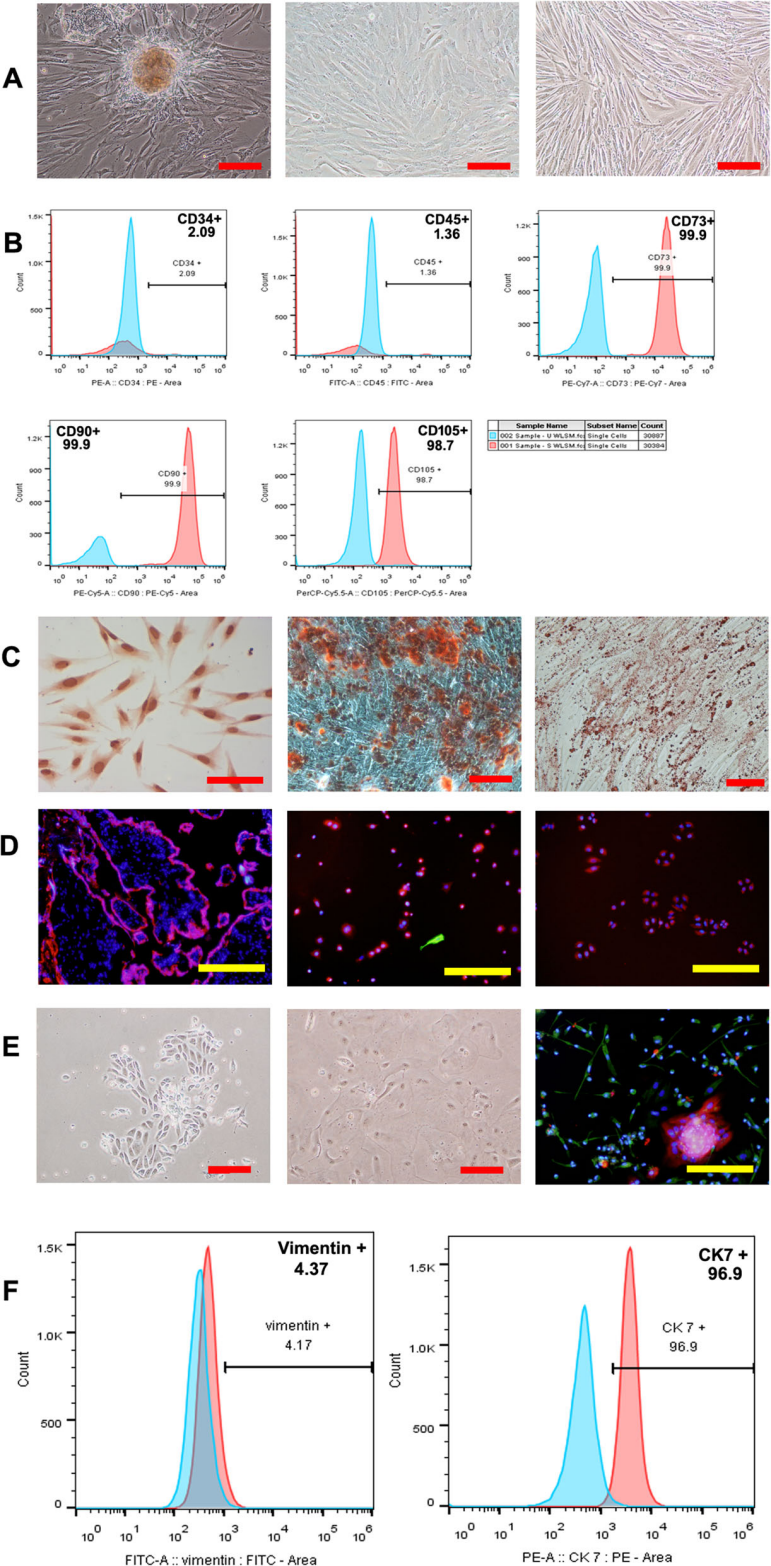
Characterization of primary early cytotrophoblast cells (early-CTBs) and primary human placenta-derived mesenchymal stem cells (hPDMSCs). **a** Morphological features of hPDMSCs isolated from human term placental tissue and passaged. The morphological features of primary hPDMSCs isolated from human full-term placental tissue (left), and the morphology features as fibroblast-like adherent cells after trypsin digestion at 0 passage (middle) and at 3rd passage (right). Scale bar 200 μm. **b** The cell surface phenotype of hPDMSCs was analyzed by flow cytometric analysis. The cells were positive for mesenchymal cell markers such as CD73, CD90, and CD105, and negative for hematopoietic cell markers such as CD34 and CD45. **c** hPDMSCs exhibited multilineage differentiation potential including endotheliocytes, osteoblasts, and adipocytes when they were cultured in the corresponding differentiation medium. The induced endothelial cells were stained with von Willebrand factor (vWF) (left), osteoblast with alkaline phosphatase (middle), and adipocytes with oil red O (right). Scale bar 100 μm. **d** Immunofluorescence was conducted to evaluate markers of cytotrophoblast cells in the placenta villus (left), the isolated primary early-CTBs (middle), and HTR8 cells line (right). Cytotrophoblast cells were stained by CK7 (red), mesenchymal cells by vimentin (green), and the cell nuclei were counterstained by 4′, 6-diamidino-2-phenylindole, dihydrochloride (DAPI) (blue). Scale bar 200 μm. **e** The early-CTBs formed multiple epithelial-like cell clones and the syncytiotrophoblast cells with the extension of the culture time. The proliferative primary CT under a light microscope (left) and the syncytiotrophoblast cells under a light microscope (middle) and immunofluorescence analyze (right). Scale bar 200 μm. **g** The markers of cytotrophoblasts were analyzed by flow cytometric analysis. The cells were positive for CK7 (cytotrophoblast marker) and negative for vimentin (mesenchymal cell marker)

It has been more than 10 years since primary human placenta-derived mesenchymal stem cells were isolated, identified, and used by our research team [19–24]. After isolation for 3–7 days, some short spindle-shaped cells, more long spindle-shaped cells, and few cobblestone-like cells gradually crawled out of the minced placental tissues and expanded to form a single clone. After being cultured for 15–21 days, the isolated hPDMSCs were confluent and passaged. Beginning at the 3rd passage (P3), the cells exhibited fibroblast-like shapes and whirlpool-clonal growth (Fig. 1a). P3 hPDMSCs were used for phenotype identification by flow cytometry and multilineage differentiation potentials. The flow cytometry analysis showed that hPDMSCs were positive for the mesenchymal stem cell markers CD73, CD90, and CD105, but negative for the hematopoietic stem cell markers CD34 and CD45 (Fig. 1b). hPDMSCs differentiated toward endotheliocytes, osteoblasts, and adipocytes in the corresponding differentiation medium. The differentiated endothelial cells were stained positive with von Willebrand factor (vWF), osteoblasts with alkaline phosphatase, and adipocytes with oil red O (Fig. 1c). Compared with middle-pregnancy and full-term placental cells, early-pregnancy MSCs exhibited shorter spindle-like shapes and had stronger proliferative abilities. However, their surface marker expression and differentiation abilities were not significantly different (data not shown).

At the same time, primary early-pregnancy cytotrophoblasts were successfully isolated by enzymatic digestion and purified by Percoll centrifugation and differential adhesion. These cells were positive for cytokeratin 7 (CK7, trophoblast marker) and negative for Vimentin (mesenchymal cell marker), similar to that of the HTR-8 cell line (Fig. 1d). After being cultured for 3–5 days, the cells expanded into multiple epithelial-like cell clones or multiple nuclear-fused syncytiotrophoblasts (Fig. 1e). However, with multiple passages, CTBs were gradually replaced with MSCs. Therefore, primary CTBs that were cultured for 1–2 days after isolation were used to collect conditioned medium for follow-up experiments. The results of flow cytometric analysis showed that these primary CTBs were positive for CK7 and negative for vimentin (Fig. 1f).

In our study, full-term placental tissue, early-pregnancy primary CTBs, and hPDMSCs from different gestational periods (early, middle, and full-term) were cultured to collect conditioned medium at different time points (placental cell-derived conditioned medium: 6, 12, 24, 48, and 72 h; sub-cultured placental tissue-derived conditioned medium: 1, 3, 5, 7, 10, and 14 days). The effect of the different conditioned media on HUVEC angiogenesis was analyzed in vitro.

### CM from placental cells or sub-cultured placental tissue promoted HUVEC proliferation

**Fig. 2:**
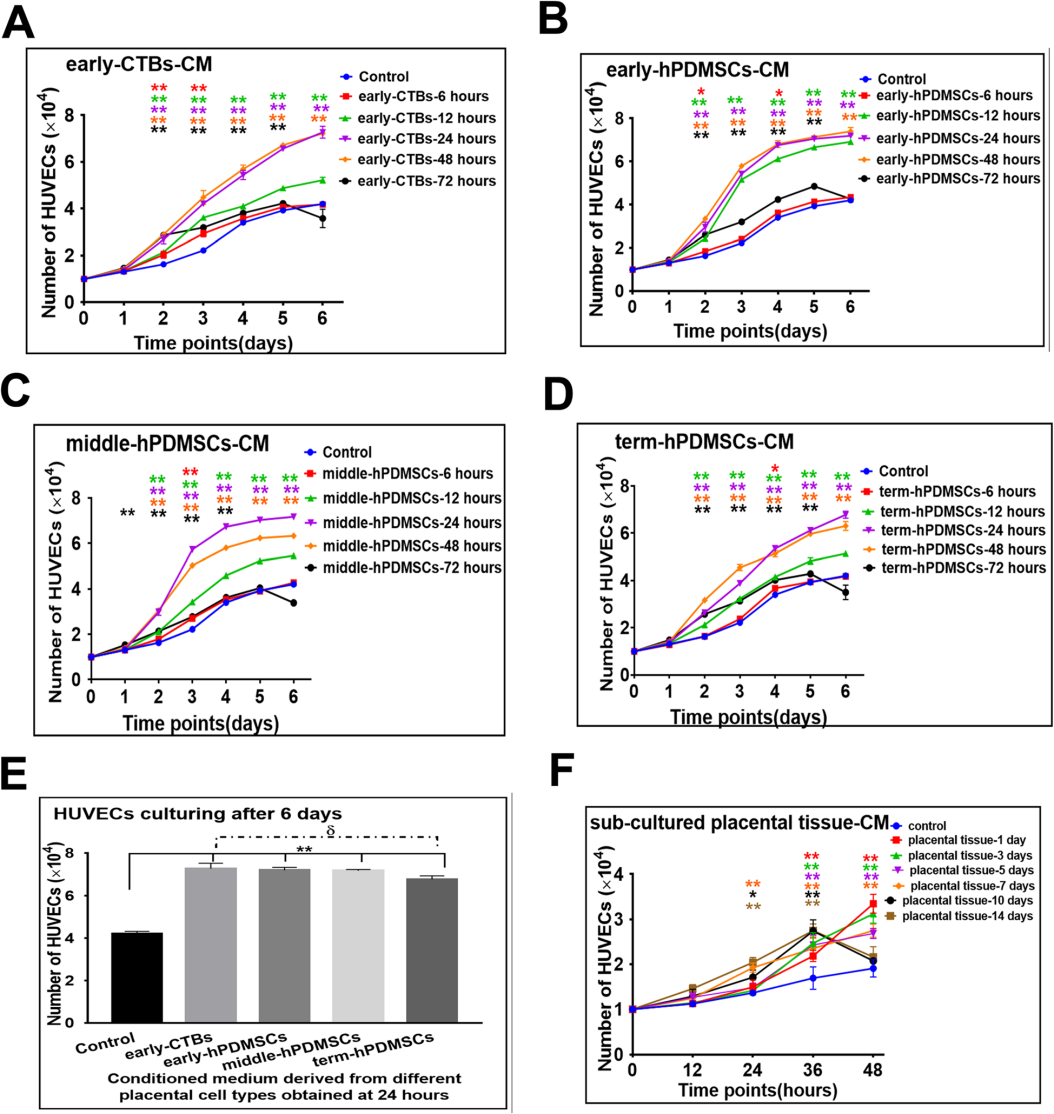
The effect of conditioned medium derived from different placental cells or sub-cultured placental tissues on the proliferation of HUVECs. **a** The growth curve on HUVECs culturing with early-CTBs-CM obtained at different time points. **b** The growth curve on HUVECs culturing with early-hPDMSCs-CM obtained at different time points. **c** The growth curve on HUVECs culturing with middle-hPDMSCs-CM obtained at different time points. **d** The growth curve on HUVECs culturing with term-hPDMSCs-CM obtained at different time points. **e** The graph of the HUVECs number on the 6th day of proliferation assay with different placental cells CM obtained at 24 h. **f** The growth curve on HUVECs culturing with sub-cultured placental tissue-CM obtained at different time points. *p < 0.05 and **p < 0.01 vs control. ^δ^*p* < 0.05 and ^δδ^*p* < 0.01 vs another group within the group

To investigate the effect of CM derived from CTBs, hPDMSCs, or sub-cultured placental tissue on HUVEC proliferation, the number of HUVECs was calculated by Countstar. The results are shown in Fig. 2.

In the placental-cells-CM groups (early-CTBs, early-hPDMSCs, middle-hPDMSCs, and term-hPDMSCs), the logarithmic growth phase appeared earlier (placental-cells-CM groups: from approximately the 2nd day; control group: from the 3rd day) and lasted longer (placental cells groups: 2 or 3 days, control group: probably 2 days) than those of the control group. From the 2nd day to the 5th day, the number of HUVECs cultured in CM from different placental cells was greater than that of cells cultured only in DMEM (control group). The effect of CM collected at different time points was different: compared with that of the control group, the effect of CM collected at 24 or 48 h on proliferation was the best, but the effect of CM collected at 6 h was not obvious. However, HUVECs exhibited slow growth, and many cell fragments were produced at the later stage of proliferation in the 72-h group (Fig. 2a–d). The number of expanded HUVECs on the 6th day of culture in 24-h CM was plotted, and the results are shown in Fig. 2e. Compared with the control group, there were significant differences in all placental cell-derived CM groups. In comparing the different placental cell types, the number of HUVECs cultured in the early-CTBs-CM group was higher than that in the term-hPDMSCs-CM group, but there was no significant difference among the remaining groups.

In the long-term sub-cultured placental tissue-CM groups, especially in the 10- and 14-day CM groups, HUVECs often showed cell cycle arrest or fragmentation in the second-half of the proliferation assay (the 3rd to the 6th day). Therefore, the number of cells was only measured in the first 2 days (Fig. 2f). The results showed that in the short-term sub-cultured placental tissue-CM groups (1-, 3-, and 5-day group), the number of HUVECs grew slowly at first and then rapidly after 24 h. Compared with that of the control group, the number of HUVECs at 36 and 48 h was significantly increased in the 1-, 3-, 5-, and 7-day sub-cultured placental tissue-CM groups. In the long-term sub-cultured placental tissue-CM groups (10 and 14 days), HUVECs grew faster in the early stage of the proliferation assay (at 12–36 h), and the cell number decreased in the later stage (at 36–48 h). The possible reason was that there were large amounts of cellular impurities or excessive metabolites in the conditioned medium from the long-term sub-cultured placental tissue.

In all conditioned medium derived from placental cells or sub-cultured placental tissue, there was no obvious effect on proliferation within 24 h, and so the experimental index was measured within 24 h in the subsequent experiments.

### CM from placental cells or sub-cultured placental tissue promoted HUVEC adhesion

**Fig. 3:**
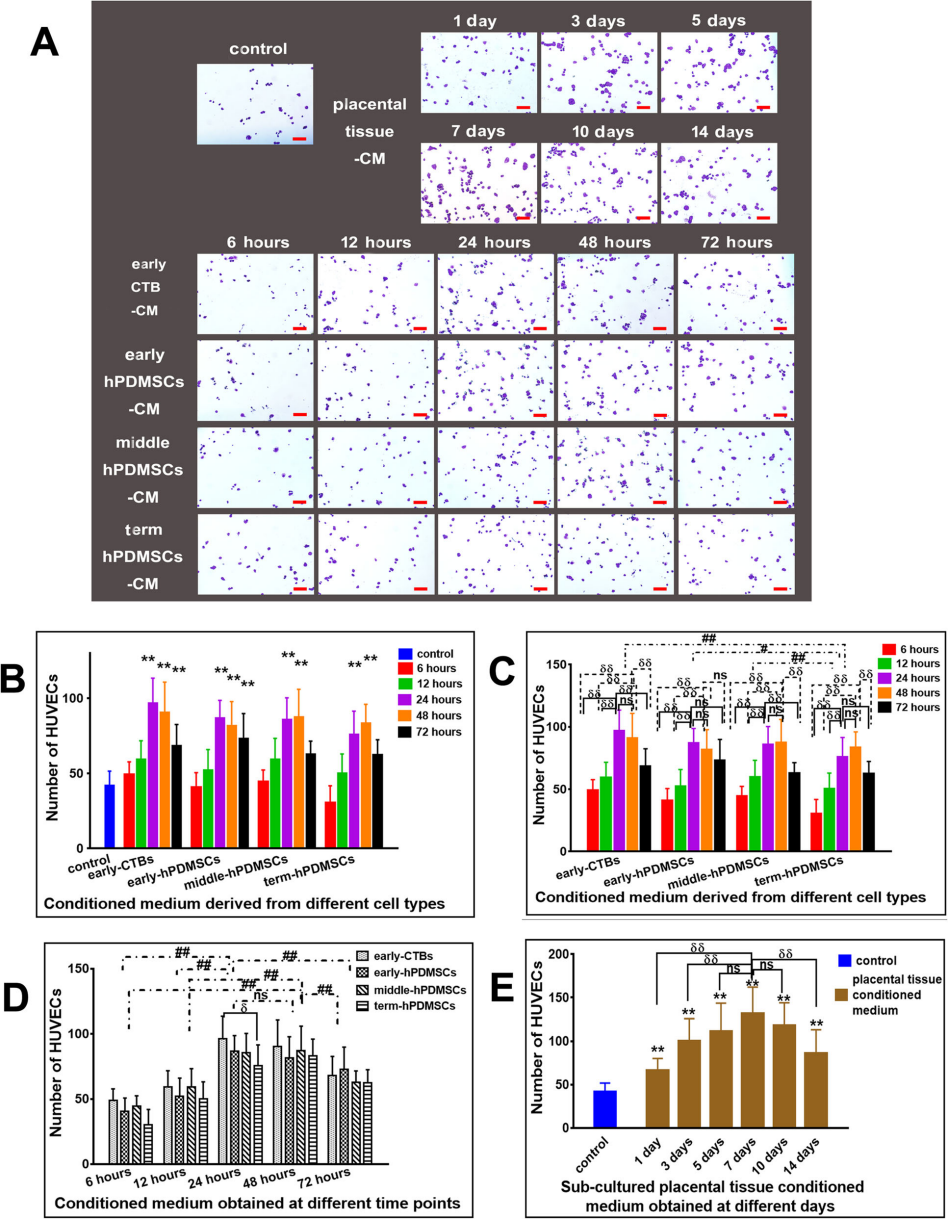
The pro-adhesive effect of conditioned medium derived from different placental cell types or sub-cultured placental tissue on HUVECs. **a** Representative images of the adherent HUVECs cultured with CM derived from different placental cell types or sub-cultured placental tissue 2 h after seeding. **b** The graph of the adhesive effect on HUVECs by CM derived from different placental cell types obtained at different time points. **c** The graph of the adhesive effect on HUVECs by CM derived from different placenta cell types (early-CTBs, early-hPDMSCs; middle -hPDMSCs, and term-hPDMSCs). **d** The graph of the adhesive effect on HUVECs by CM obtained at different time points (6, 12, 24, 48, and 72 h). **e** The graph of the adhesive effect on HUVECs by CM derived from sub-cultured placental tissue obtained at different time points (1, 3, 5, 7, 10, and 14 days). *p < 0.05 and **p < 0.01 vs control. ^δ^*p* < 0.05 and ^δδ^*p* < 0.01 vs another group within the group. ^#^*p* < 0.05 and ^##^*p* < 0.01 vs another group between groups, *ns* indicates no significant difference

In the adhesion assay, the number of adherent HUVECs was measured after culture for 2 h. The results suggested that, compared with that of the control group, the number of adherent cells increased in all groups treated with placenta cell-derived CM collected at 24 and 48 h. Among the 72-h groups, only early-hPDMSCs-CM and early-CTBs-CM enhanced adhesion, as shown in Fig. 3b.

The graphical analysis of the different placental cell types as the abscissa is shown in Fig. 3c. Among the different placental cell type groups, the adhesion-promoting effect of the term-hPDMSCs-CM group was weaker than that of the remaining three cell type groups, but there was no significant difference among these three groups. Within each cell type, CM collected at 24 and 48 h had the best adhesion-promoting effect, which was almost higher than that of the other time points (except the 24- and 72-h groups of early-hPDMSCs-CM and the 48- and 72-h term-hPDMSCs-CM groups).

The CM that was collected at different time points was used as the abscissa for plot analysis (Fig. 3c). The results showed that CM collected at 24 and 48 h was better than that collected at other time points, but there was no significant difference between them. Within each time point, there was no significant difference among the four placental cell types at the 48 h, but the effect of early-CTBs-CM was better than that of term-hPDMSCs-CM at the 24 h.

In the sub-cultured placental tissue groups, compared with the control group, short-term tissue culture groups and long-term tissue culture groups had stronger adhesion-promoting effects. The effect of the 7-day group was the most significant, which was better than that of the 1-, 3-, or 14-day groups, but was not obvious compared with that of the 5- or 10-day groups (Fig. 3e).

### CM from placental cells or sub-cultured placental tissue promoted HUVEC migration

**Fig. 4:**
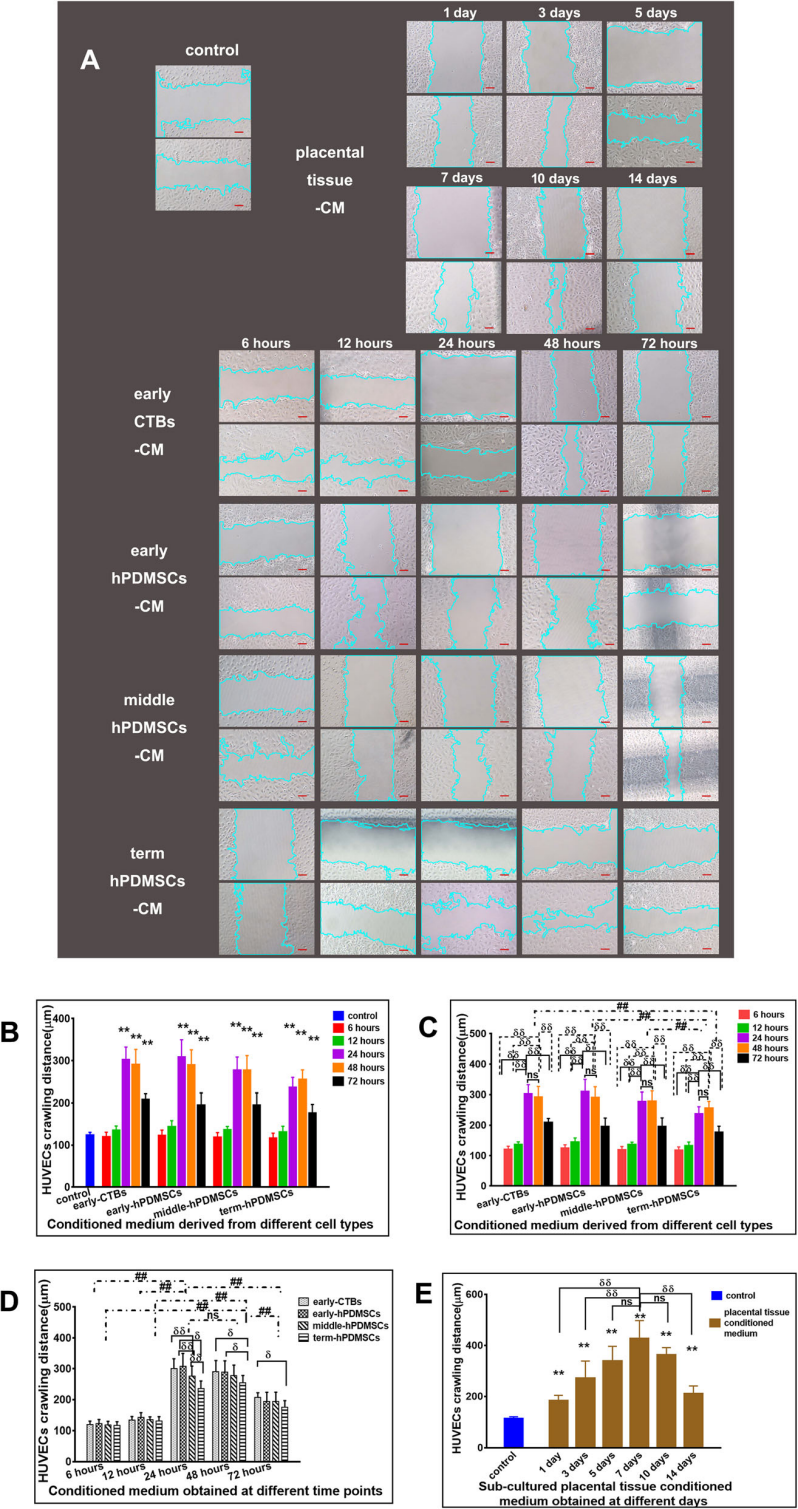
The effect of CM derived from placental cells or sub-cultured placental tissue on the horizontal migration of HUVECs in wound healing assay. **a** Representative images of HUVECs both at 0 h and incubated for 8 h with CM derived from different placental cell types or sub-cultured placental tissue in wound healing assay. **b** The quantitative assessment of the promoting horizontal migration effect on HUVECs by CM derived from different placental cell types obtained at different time points. **c** The graph of the promoting horizontal migration effect on HUVECs by CM derived from different placenta cell types (early-CTBs, early-hPDMSCs; middle-hPDMSCs, and term-hPDMSCs). **d** The graph of the promoting horizontal migration effect on HUVECs by CM obtained at different time points (6, 12, 24, 48, and 72 h). **e** The graph of the promoting horizontal migration effect on HUVECs by CM derived from sub-cultured placental tissue obtained at different time points (1, 3, 5, 7, 10, and 14 days). *p < 0.05 and **p < 0.01 vs control. ^δ^*p* < 0.05 and ^δδ^*p* < 0.01 vs another group within the group. ^#^*p* < 0.05 and ^##^*p* < 0.01 vs another group between groups, *ns* indicates no significant difference

In the scratch wound healing assay, the cell horizontal migration distance was measured. The results are shown in Fig. 4.

All placenta cell-derived CM collected at 24, 48, and 72 h increased the closure distance of HUVECs compared with that of the control medium (Fig. 4a, b).

Figure 4 shows the plot analysis with the different placental cell types as the abscissa. Comparative analysis between the different placental cell type groups revealed that the migration-promoting effect of term-hPDMSCs-CM was weaker than that of the other three groups, but there was no significant difference among them. Within each placental cell type group, 24 and 48 h had the best promigratory effect, but there was no difference among them (Fig. 4c).

CM collected at different time points was used as the abscissa to analyze the variables. The results showed that the CM of all placental cell types collected at 24 and 48 h was better than that collected at other time points, but there were no differences between them. Within each time point group, in the 24-h group, the term-hPDMSCs-CM group had a weaker effect than the other three groups, and the early-hPDMSCs-CM group has a better effect than the middle-hPDMSCs-CM group, but there was no significant difference among the other groups. In the 48-h groups, early-CTBs and early-hPDMSCs were better than term-hPDMSCs (Fig. 4d).

The sub-cultured placental tissue-derived CM that was collected at all the time points (1, 3, 5, 7, 10, and 14 days) had better promigratory effects than that of the control group. The 7-day group had the most robust effect, which was significantly better than that of the 1-, 3-, and 14-day groups, but there was no significant difference compared with that of the 5- and 10-day groups (Fig. 4e).

**Fig. 5:**
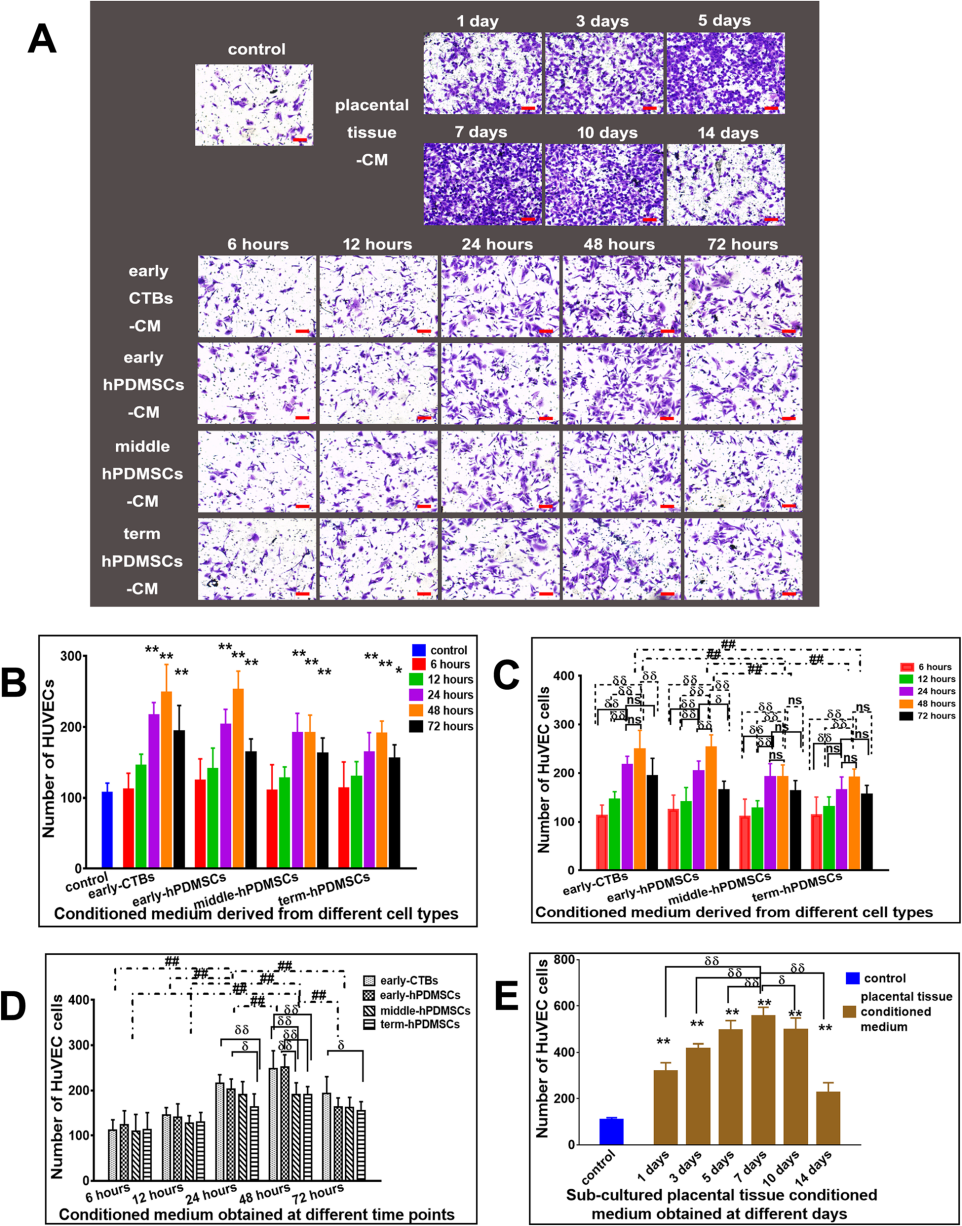
The effect of CM derived from placental cells or sub-cultured placental tissue on the vertical migration of HUVECs in transwell migration assay. **a** Representative images of migrated HUVECs incubated for 18 h with CM derived from different placental cell types or sub-cultured placental tissue in transwell migration assay. **b** The quantitative assessment of the promoting vertical migration effect on HUVECs by CM derived from different placental cell types obtained at different time points. **c** The graph of the promoting vertical migration effect on HUVECs by CM derived from different placental cell types (early-CTBs, early-hPDMSCs; middle-hPDMSCs, and term-hPDMSCs). **d** The graph of the promoting vertical migration effect on HUVECs by CM obtained at different time points (6, 12, 24, 48, and 72 h). **e** The graph of the promoting horizontal migration effect on HUVECs by CM derived from sub-cultured placental tissue obtained at different time points (1, 3, 5, 7, 10, and 14 days). *p < 0.05 and **p < 0.01 vs control. ^δ^*p* < 0.05 and ^δδ^*p* < 0.01 vs another group within the group. ^#^*p* < 0.05 and ^##^*p* < 0.01 vs another group between groups, *ns* indicates no significant difference

In Transwell migration assay, the migrated cells were counted. The results are shown in Fig. 5.

Compared with that of control CM, CM collected from all placental cells at 24, 48, and 72 h increased the number of migrated HUVECs (Fig. 5b).

Using the different placental cell types as the abscissa, the graphical analysis is shown in Fig. 5c. Comparative analysis of the different placental cell types revealed that the promigratory effects of the early-CTBs-CM and early-hPDMSCs-CM groups were stronger than those of the middle-hPDMSCs-CM and term-hPDMSCs-CM groups. However, there was no significant difference among the early-placental cell-derived CM groups, and there was no difference between middle- and term-hPDMSCs-CM groups. Comparative analysis within the groups and among all four placental cell types showed that the promotion of vertical migration by CM obtained at 24 and 48 h was robust (only the 48-h group had the best effect among the term-hPDMSCs-CM groups), and there was no significant difference between them (except 24 h, which was better than 48 h in the early-hPDMSCs-CM groups). Compared with CM at other time points, both 24 h and 48 h were stronger than most of the other groups (except that in the early-CTBs-CM, middle-hPSMSCs-CM and term-hPDMSCs-CM groups, there was no difference between 24 h and 72 h, and in the middle-hPDMSCs-CM and term-hPDMSCs-CM groups, there was no difference between 48 h and 72 h) (Fig. 5c).

The CM collected at different time points was used as the abscissa to analyze the variables. The results are shown in Fig. 5d. Conditioned medium collected at 48 h had the best effect among the different time points. Comparative analysis within each time point group showed that in the 24-h CM group, the promigratory effects of early-CTBs-CM and early-hPDMSCs-CM were better than that of term-hPDMSCs-CM; in the 48-h CM group, the effects of early-CTBs-CM and early-hPDMSCs-CM were stronger than those of middle-hPDMSCs-CM and term-hPDMSCs-CM; and in the 72-h CM group, early-CTBs-CM was better than term-hPDMSCs-CM (Fig. 5d).

In the sub-cultured placental tissue-derived CM groups, compared with the control group, all groups had stronger effects on promoting migration, and the effect of the 7-day group was the strongest (Fig. 5e).

### CM from placental cells or sub-cultured placental tissue promoted HUVEC invasion

**Fig. 6:**
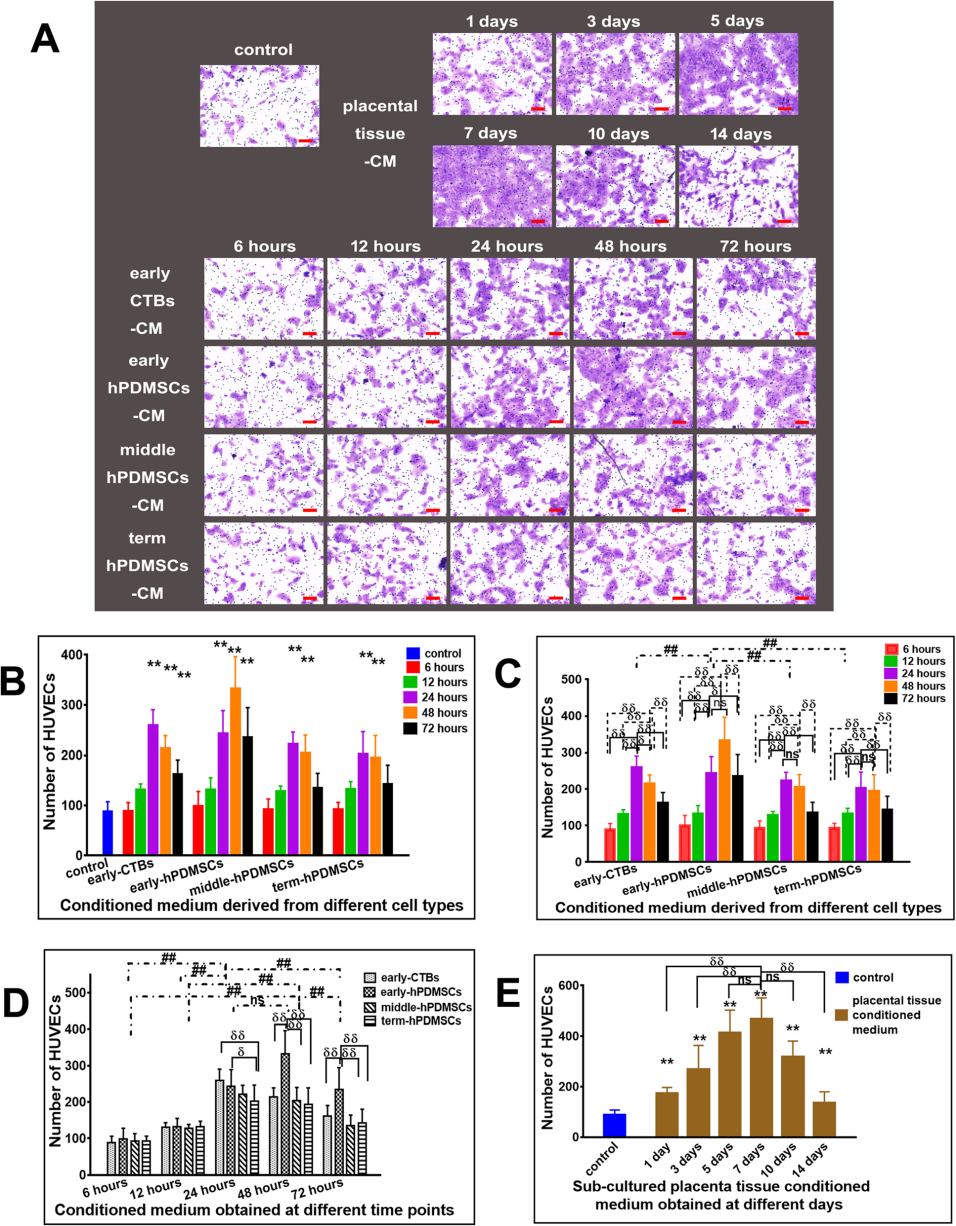
The effect of CM derived from placental cells or sub-cultured placental tissue on the invasion ability of HUVECs in transwell invasion assay. **a** Representative images of invading HUVECs incubated for 18 h with CM derived from different placental cell types or sub-cultured placental tissue in transwell invasion assay. **b** The quantitative assessment of the promoting invasion effect on HUVECs by CM derived from different placental cell types obtained at different time points. **c** The graph of the promoting invasion effect on HUVECs by CM derived from different placental cell types (early-CTBs, early-hPDMSCs; middle-hPDMSCs, and term-hPDMSCs). **d** The graph of the promoting invasion effect on HUVECs by CM obtained at different time points (6, 12, 24, 48, and 72 h). **e** The graph of the promoting invasion effect on HUVECs by CM derived from sub-cultured placental tissue obtained at different time points (1, 3, 5, 7, 10, and 14 days). *p < 0.05 and **p < 0.01 vs control. ^δ^*p* < 0.05 and ^δδ^*p* < 0.01 vs another group within the group. ^#^*p* < 0.05 and ^##^*p* < 0.01 vs another group between groups, *ns* indicates no significant difference

In Transwell invasion assay, the invasive HUVECs were analyzed. The results are shown in Fig. 6c.

Compared with the control group, placental cell-derived CM collected at 24 and 48 h increased the number of cells that degraded the Matrigel and moved through the membrane. CM derived from both early-CTBs and early-hPDMSCs that were collected at 72 h also enhanced the invasive effect (Fig. 6b).

Using the different placental cell types as the abscissa, the graphical analysis is shown in Fig. 6c. Comparative analysis between the different placental cell types revealed that the number of invasive HUVECs in the early-hPDMSCs-CM group was the highest among all the CM groups, and there was no significant difference among the other three groups. Comparative analysis within each group showed that the invasion-promoting effect of CM collected at 24 h was the strongest in the early-CTBs-CM group. In the three hPDMSCs-CM groups, the effect of CM collected at 24 and 48 h was better than that of CM collected at other time points; moreover, there was no significant difference between 24 and 48 h (Fig. 6c).

The different time points were used as the abscissa for plot analysis. The results are shown in Fig. 6d. The effect of CM collected at 24 and 48 h was the best among the different time points, and there was no significant difference between 24 and 48 h. The intragroup comparison showed that in the 24-h group, the effects of early-CTBs-CM and early-hPDMSCs-CM were better than that of term-hPDMSCs-CM. However, in the 48- and 72-h groups, early-hPDMSCs-CM was the best among the four placental cell type groups (Fig. 6d).

In the sub-cultured placental tissue-derived CM groups, compared with the control group, short-term culture groups and long-term culture groups had stronger effects on promoting invasion. The 7-day CM had the strongest effect, which was significantly better than that of 1-, 3-, or 14-day CM, but there was no significant difference compared with that of 5- or 10-day CM (Fig. 6e).

### The angiogenic effect from CM of placental cells or sub-cultured placental tissue

**Fig. 7:**
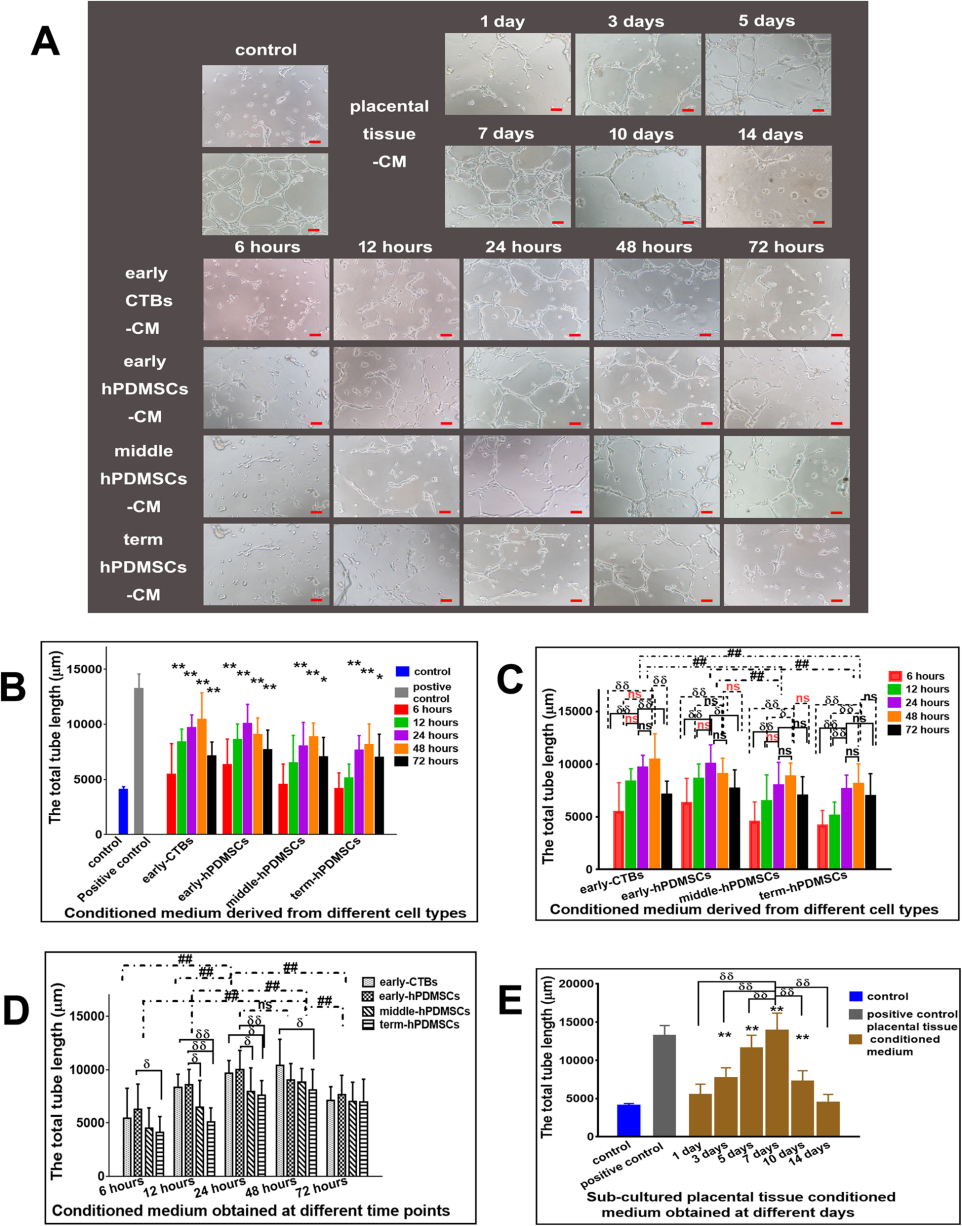
The proangiogenic effect of CM derived from placental cells or sub-cultured placental tissue in Matrigel tube formation assay. **a** Representative images of the capillary-like tube structures of HUVECs incubated for 15 h with CM derived from different placental cell types or sub-cultured placental tissue in Matrigel tube formation assay. **b** The quantitative analysis of the total length of tube formation of HUVECs by CM derived from different placental cell types obtained at different time points. **c** The graph of the angiogenic effect on HUVECs by CM derived from different placental cell types (early-CTBs, early-hPDMSCs; middle-hPDMSCs, and term-hPDMSCs). **d** The graph of the angiogenic effect on HUVECs by CM obtained at different time points (6, 12, 24, 48, and 72 h). **e** The graph of the angiogenic effect on HUVECs by CM derived from sub-cultured placental tissue obtained at different time points (1, 3, 5, 7, 10, and 14 days). *p < 0.05 and **p < 0.01 vs control. ^δ^*p* < 0.05 and ^δδ^*p* < 0.01 vs another group within the group. ^#^*p* < 0.05 and ^##^*p* < 0.01 vs another group between groups, *ns* indicates no significant difference

In tube formation assay, the total length of the tube was quantified using ImageJ software. As shown in Fig. 7a, compared with the negative control CM derived from the four placental cell types at 24, 48, and 72 h had obvious proangiogenic effects, and at 12 h, only CM from the early-CTBs and early-hPDMSCs promoted angiogenesis (Fig. 7b).

The results of the graphical analysis with the different placental cell types as the abscissa are shown in Fig. 7c. Comparative analysis between the different placental cell types revealed that the total tube lengths of HUVECs incubated in early-CTBs-CM and early-hPDMSCs-CM were longer than those incubated in middle-hPDMSCs-CM and term-hPDMSCs-CM, and there was no significant difference between the early-CTBs-CM and early-hPDMSCs-CM groups or between the middle-hPDMSCs-CM and term-hPDMSCs-CM groups. Comparative analysis within the groups showed that the angiogenic effects of 24- and 48-h CM were stronger than those of CM collected at the other time points among the placental cell types, although the differences among some groups were not statistically significant (Fig. 7c).

The results of the plot analysis, using different time points as the abscissa, are shown in Fig. 7d. Comparative analysis between the different time points showed that the promoting effect of 24- and 48-h CM was strongest among the time points, but there was no significant difference between them. Comparative analysis within each time point showed that in the 12- and 24-h groups, the effects of early-CTBs-CM and early-hPDMSCs-CM were stronger than that of term-hPDSMCs-CM, and early-hPDMSCs-CM was stronger than middle-hPDMSCs-CM. In the 48-h group, the effect of early-CTBs-CM was better than that of term-hPDMSCs-CM (Fig. 7d).

In the in vitro Matrigel tube formation assay with sub-cultured placental tissue-derived CM, compared with that of the control group, there was an obvious proangiogenic effect in the 3-, 5-, 7-, and 10-day sub-cultured placental tissue-derived CM groups. The 7-day CM group had the most significant proangiogenic effect of all the CM groups (Fig. 7e). The results were the same as those for adhesion, migration, and invasion.

### Angiogenic factor expression in placental cells or sub-cultured placental tissue

**Fig. 8:**
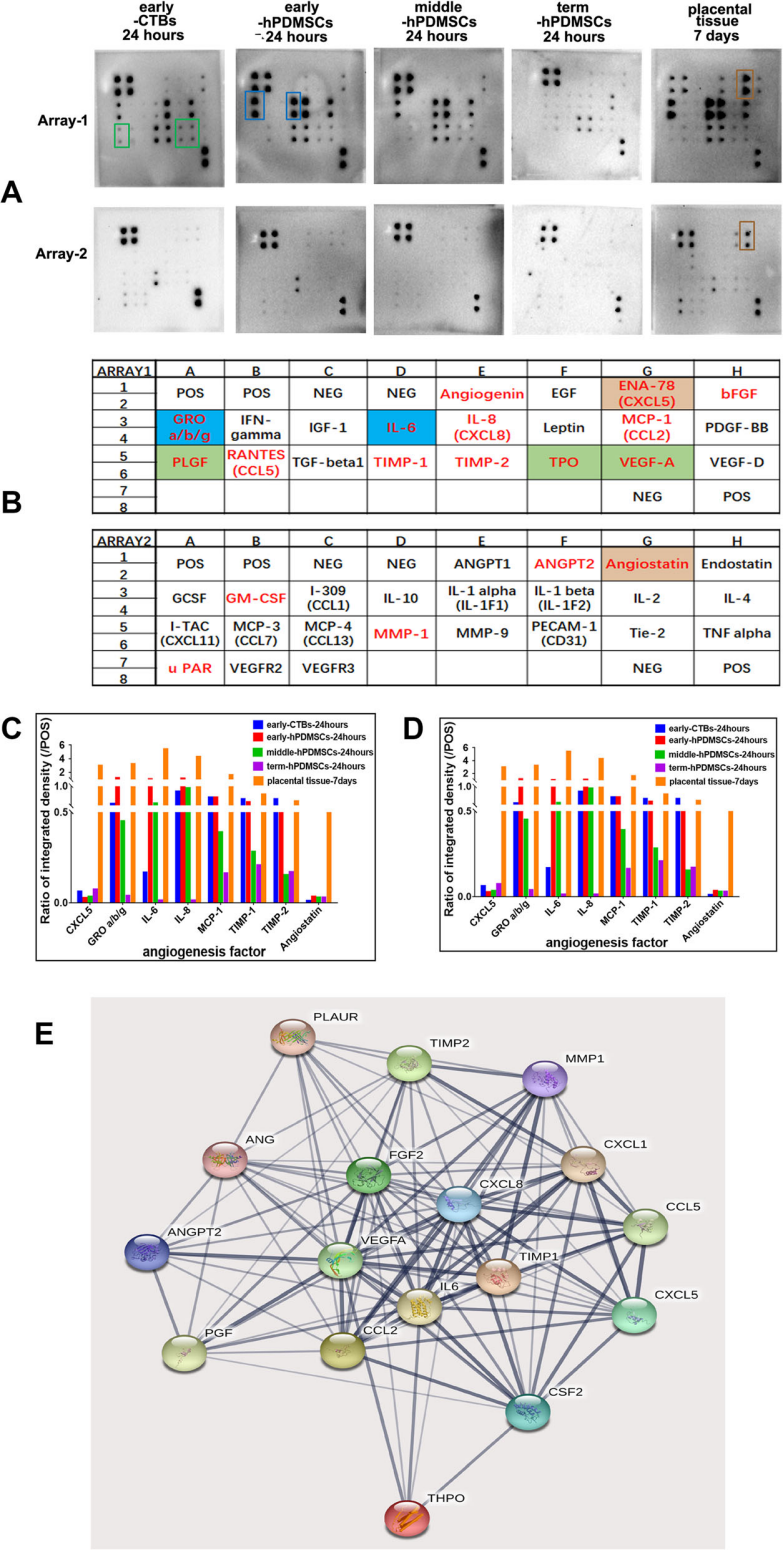
Angiogenic factor expression of placental-cell-derived CM or sub-cultured placental tissue-derived CM in human angiogenesis antibody array. **a** Representative images of the content of 43 conventional angiogenic factors in the CM of four types of placental cells cultured for 24 h or the CM of sub-cultured placental tissue cultured for 7 days were detected by the human angiogenesis antibody array. **b** The layout of 46 conventional angiogenic factors in angiogenesis antibody array membrane. **c**, **d** The histogram of the expression of angiogenic factors in the CM of four types of placental cell cultured for 24 h or the CM of placental tissue cultured for 7 days. **e** The analysis of protein-protein interaction of secreted proteins of the placenta. All these 17 factors could interact with others and the thickness of the connecting lines represents the strength of the interaction between factors. The red font represents all detected angiogenic factors in the placenta. The brown box represents the high content of this angiogenic factor in sub-cultured placental tissue-CM, the green box represents the high content of this angiogenic factor in early-CTBs-CM, and the blue box represents the high content of this angiogenic factor in hPDMSCs-CM

According to our results, placental cell-derived or sub-cultured placental tissue-derived CM can promote HUVEC proliferation, adhesion, migration, invasion, and tube formation to varying degrees, thus enhancing angiogenesis. The effects of 24- and 48-h placental cell-derived CM or 7-day sub-cultured placental tissue-derived CM were the strongest. To further investigate the mechanism, the levels of 43 conventional angiogenic factors in 24-h placental cell-derived CM and in 7-day sub-cultured placental tissue-derived CM were measured with a human angiogenesis antibody array (RayBiotech, USA). The results are shown in Fig. 8.

Among these 43 factors, in sub-cultured placental tissue-derived CM, the 5 factors with the highest levels were GRO, IL-6, IL-8, CXCL-5, and MCP-1 (ratio to the positive control: > 1); the levels of the following 3 factors were moderate: TIMP-1, TIMP-2, and angiostatin (0.5~1); and the ratios of the following 6 factors ranged from 0.1 to 0.5: angiogenin, bFGF, TPO, VEGF-A, MMP-1, and uPAR. In addition, the ratios of 4 factors ranged from 0.1 to 0.05 (PLGF, CCL5, ANGPT2, and GM-CSF). Among these 18 factors, the levels of CXCL-5 and angiostatin were increased in sub-cultured placental tissue-derived CM but were low or undetectable in hPDMSCs-CM or early-CTBs-CM. The levels of other factors in sub-cultured placental tissue-derived CM were significantly higher than those of placental cell-derived CM (except MMP-1 and PLGF). Among the different placental cell types, the levels of PLGF, TPO, and VEGF-A were higher in early-CTBs-CM, while the levels of GRO and IL-6 in early-hPDMSCs-CM or middle-hPDMSCs-CM were significantly higher than those in early-CTBs-CM and term-hPDMSCs-CM. IL-8, MCP-1, TIMP-1, TIMP-2, angiogenin, bFGF, MMP-1, uPAR, CCL5, ANGPT2, and GM-CSF were all present in both early-CTBs-CM and hPDMSCs-CM, but the concentrations of these factors in placental cells need to be further confirmed (Fig. 8a–d). The protein-protein interactions of these 17 secreted factors in the placenta (except for angiostatin) were analyzed by STRING11.0. In the graph, some factor names were different (‘bFGF’: FGF2; ‘GRO’: CXCL1; ‘IL-8’: CXCL8; ‘MCP-1’: CCL2; ‘PLGF’: PGF; TPO-THPO: thrombopoietin; ‘GMCSF’: CSF2; ‘uPAR’: PLAUR). The interaction network is shown in Fig. 8e. The thickness of the connecting lines represented the strength of the interaction between the factors. All 17 of these factors were related to each other, and multiple proteins had multiple action points associated with cell activity. These results can provide ideas for subsequent experiments.

## Discussion

Placental blood vessel formation includes vasculogenesis and angiogenesis, which begins as early as day 21 of pregnancy and continues throughout gestation.

The formation process, whether vasculogenesis or angiogenesis, is strictly regulated and overlaps to a certain extent [4, 29, 39]. Throughout pregnancy, angiogenic factors produced by placental cells play key roles in placental blood vessel development. In the placental milieu, placental mesenchymal stem cells and trophoblasts may interact with placental endothelial cells through autocrine and paracrine mechanisms to regulate the normal development of the placental vascular network [8, 40].

Therefore, combined with our team’s more than 10 years of experiences with primary hPDMSC isolation and applications, the two most abundant types of placental cells, primary cytotrophoblasts and primary mesenchymal stem cells (especially hPDMSCs from different pregnancy periods), were used as the cell research objects, and postpartum placental tissues were used as the tissue research object to explore their regulation of placental vascular development during different pregnancy periods. In our study, primary early CTBs were successfully obtained, but these cells were gradually replaced by hPDMSCs during passage. Therefore, primary CTBs, which were cultured 1–2 days after primary isolation, were used to collect conditioned medium for follow-up experiments.

We examined the effect of placental cell- or tissue-derived CM on HUVEC proliferation, adhesion, horizontal and vertical migration, invasion, and tube formation in vitro, then detected the contents of 43 angiogenic factors in the CM. The main results were as follows: (i) primary placental cell-derived CM promoted a series of angiogenic process in vitro to some extent, (ii) the effects of primary early-pregnancy CTBs or hPDMSCs in promoting angiogenesis were stronger than hPDMSCs from full-term pregnancy, (iii) the sub-cultured placental tissue-derived CM promoted angiogenesis in vitro, (iv) among the different time points, placental cell-derived CM collected at 24 h or 48 h had the best effect and sub-cultured placental tissue-derived CM collected at 7 days had the best effect, and (v) the semiquantitative angiogenesis antibody array showed that in placental cell-derived CM or sub-cultured placental tissue-derived CM, 18 of the 43 angiogenic factors had obvious spots and the levels of 5 factors were the highest.

Consistent with the results of previous studies, our results showed that not only early-CTBs but also hPDMSCs promoted a series of angiogenic processes in HUVECs to some extent. Many scholars have found that different types of cytotrophoblasts promote angiogenesis, which was similar to our results [6, 41–44]. For example, purified primary cytotrophoblasts were used by Knöfler or Kato et al., the choriocarcinoma-derived BeWo cell line was used by Troja et al., and the normal human first-trimester extravillous cytotrophoblast (evCTB)-derived HTR-8 cell line was used by Kalkunte or Das et al. For hPDMSCs, our conclusion was also consistent with that of many studies; hPDMSCs obviously contribute to a series of cell activities to promote angiogenesis [9–13, 15]. We further compared the effect of these two types of placental cells and found that primary placental cells derived from early pregnancy, whether CTBs or hPDMSCs, had more obvious effects in promoting angiogenesis than hPDMSCs from full-term pregnancy. Among these cell activities, there was no significant difference in the effect on proliferation among the four kinds of cells (only early-CTBs and early-hPDMSCs had a slight difference, *p* < 0.05). In the adhesion assay and wound healing assay, the effect of term-hPDSMCs was weaker than that of the other three cell types (Fig. 3c and Fig. 4c). In the in vitro transwell migration assay and tube formation experiment, the effects of early-CTBs and early-hPDMSCs were better than those of middle-hPDMSCs and term-hPDMSCs, but in the invasion assay, only early-hPDMSCs were better than other cell types (Figs. 5c, 6c, and 7c).

In our study, we evaluated the effect not only of placental cells but also sub-cultured placental tissue on placental angiogenesis. In the proliferation experiment, although the extracted tissue-derived CM was sterilized by filtration and other means, massive cell death occurred when HUVECs were cultured for a long time. The reason may be the high concentration of metabolites. Therefore, only 2 days of proliferation was measured, the effect of sub-cultured placental tissue-derived CM on proliferation occurred earlier (36 h), and the effect of 7-day sub-cultured placental tissue-derived CM was the strongest. Moreover, sub-cultured placental tissue-derived CM also strongly promoted angiogenesis in a series of experiments, and the effect of 7-day sub-cultured placental tissue-derived CM was the strongest (Figs. 3, 4, 5, 6, and 7e). At the same time, the results showed that the effect of sub-cultured placental tissue-derived CM was stronger than that of CM derived from individual placental cell types. The possible reasons are as follows. First, the compositions of them are different. There are multiple cell types in sub-cultured placental tissue-derived CM, such as trophoblasts, MSCs, Hofbauer cells, vascular smooth muscle cells, perivascular cells, and endothelial cells, which play an important role in placenta angiogenesis [4]. Second, the volume of placental tissue for tissue-derived CM and primary single cell-derived CM is different. Third, the cultured time to collect them is also different. So their comparability needs to be further verified.

Experiments using conditioned medium from cells or tissue are a recognized research method. Conditioned medium is the spent medium after cells are cultured. It contains various growth factors, metabolites, and ECM secreted into the culture medium by the cultured cells. Because of the variety of cell sources, the various isolation methods of primary cells or different methods of collecting conditioned medium, the effects may be different. In addition, it is unknown whether longer culture times result in more robust effects. A study by Chang used different time points, including 6, 12, 24, 48, and 72 h for placental cell-derived CM [45] and 1, 3, 5, 7, 10, and 14 days for sub-cultured placental tissue-derived CM, to achieve optimal effects. The results showed that the optimal time points for placental cell-derived CM were 24 and 48 h but not 72 h. There was almost no significant difference between 24 and 48 h in a series of angiogenesis experiments (except for the transwell migration assay). Although there was no significant difference, the effects of 24-h CM from early-placental cells and middle-hPDMSCs were better than those of CM from other time points, and the effect of 48-h CM from term-hPDMSCs was better. Therefore, we used 24-h CM derived from placental cells for the angiogenic factor array. Among the CM of sub-cultured placental tissue collected at different days, the angiogenic effect was the best at 5 and 7 days but not at 14 days. In addition, the vascular-like structures formed by CM at 10 and 14 days, especially at 14 days, were obviously unhealthy. Therefore, 7-day sub-cultured placental tissue-derived CM was used for future experiments.

To further explore the mechanism of placental cell and sub-cultured placental tissue angiogenesis, we measured the levels of 43 angiogenic factors with a semiquantitative angiogenesis protein array, which further verified our results. The levels of angiogenic factors in full-term-pregnancy hPDMSCs-CM at 48 h were higher than those at 24 h, while those in early-CTBs-, early-pregnancy hPDMSCs-, and middle-pregnancy hPDMSCs-CM were not obviously different (data not shown). The angiogenic factor levels of 24-h placental cell-derived CM and 7-day sub-cultured placental tissue-derived CM are shown in Fig. 8. The levels of angiogenic factors in sub-cultured placental tissue-derived CM were the highest; furthermore, the levels of factors in early-hPDMSCs-CM were higher than those in full-term-hPDMSCs-CM. This was consistent with our results: 7-day sub-cultured placental tissue-derived CM had the strongest angiogenic effect and the effect of early-pregnancy placental cells was stronger than that of full-term-pregnancy placental cells.

Among these 43 angiogenic factors, 18 factors had obvious spots in sub-cultured placental tissue-derived CM, and the ratios of their levels to the positive control were > 0.05, as shown in Fig. 8 c and d. These 18 angiogenic factors included GRO, IL-6, IL-8, CXCL-5, MCP-1, TIMP-1, TIMP-2, angiostatin, angiogenin, bFGF, TPO, VEGF-A, uPAR, CCL5, ANGPT2, GM-CSF, PLGF, and MMP-1. Four other angiogenic factors also had visible spots, but their levels were very low (including CCL13, CD31, Tie-2, VEGFR2, and VEGFR3; data not shown). The levels of 18 factors in sub-cultured placental tissue were significantly higher than those of any placental cell type in our study, except for PLGF and MMP-1. The level of PLGF in early-CTBs was higher than that in other placental cell groups, which was consistent with the report showing that PLGF is mainly produced by cytotrophoblasts. In the sub-cultured placental tissue-derived CM, the factors with the highest levels were CXCL-5, GRO, IL-6, IL-8, and MCP-1. This result was similar to those of studies by DU [31] and other groups [12, 14, 31, 46–48]. The level of CXCL5 was very low in hPDMSCs-CM and early-CTBs-CM but high in sub-cultured placental tissue-derived CM, indicating that CXCL5 may be secreted by other placental cells. Among the remaining highest factors, the levels of IL-8 and MCP-1 were higher in both hPDMSCs-CM and early-CTBs-CM, and the levels of IL-6 and GRO in hPDMSCs-CM were significantly higher than those in early-CTBs-CM. In addition to these 5 highest factors, 10 other angiogenic factors in placental cells or sub-cultured placental tissue were also increased, and the levels of PLGF, TPO, and VEGF-A in early-CTBs were significantly higher than those in hPDMSCs. These results suggest that hPDMSCs and CTBs play important roles in regulating placental angiogenesis, but the degree and mechanism of factor secretion need to be further studied. TIMP-1, TIMP-2, and angiostatin are antiangiogenic factors that balance placental vascular development. Moreover, our results showed that the levels of the VECF family factors, VEGF-A, VEGF-D, and PLGF were far lower than those of CXCL5, GRO, IL-6, IL-8, and MCP-1. Although some scholars have obtained the same results [12, 31], this finding is different from the widely recognized view that VEGF family factors are the most critical factors in placental angiogenesis regulation [2, 3, 5–7]. Because this angiogenesis factor array does not contain all known angiogenic factors, whether VEGF family factors or other unexplored angiogenic factors play roles in placental angiogenesis remains to be further verified. Based on the levels of angiogenic factors, we also used STRING 11.0 to predict the interactions of these 17 angiogenic factors (Fig. 8e), which provided directions for further experiments.

In addition to helping to further research on the mechanism of placental angiogenesis regulation, this study is also relevant to vascular tissue engineering and clinical treatments.

In addition to their differentiation potential, hPDMSCs also have strong secretory abilities and have been widely used in many disease treatments, both in animal models and clinical patients, such as in wound healing, ischemic heart disease, chronic lung injury, diabetes, ankylosing spondylitis, and myelodysplasia [31, 48, 49]. However, while MSCs can be injected into the body, the low viability, potential immunogenicity, or even tumorigenicity of implanted hPDMSCs in recipients undermines the efficacy and safety of this cell-based treatment and hampers its widespread clinical application. In our study, hPDMSCs-CM contained abundant levels of angiogenic factors and had a proangiogenic effect to a certain extent, and so it would have not only a reduced risk compared to that of cell infusion but also have a certain therapeutic effect. Furthermore, sub-cultured placental tissue-derived CM, similar to placental extract, has also been used in many disease treatments in recent years, such as in ischemic diseases, in scaffolds for engineered tissue [50], to treat osteoarthritis [51], for regenerating sciatic nerves [52], and in burn injuries, chronic ulcers, skin defects [53, 54], climacteric symptoms [55], and hair loss [56]. These uses are mainly associated with the biological properties of the sub-cultured placental tissue, such as proangiogenic effects, wound protection [53, 54], anti-inflammatory and antioxidative effects, anti-platelet aggregation activity [57, 58], anti-aging, and low immunogenicity. The most fundamental reason is that the placenta contains abundant extracellular matrix (ECM) components and bioactive molecules [50, 59]. In our study, the angiogenic effect of sub-cultured placental tissue-derived CM and its proteomics were also examined, and the results suggested that compared with hPDMSCs-CM, sub-cultured placental tissue-derived CM had a stronger angiogenic effect and higher angiogenic factor levels. The effect of these angiogenic factors was different. IL-8 (also known as CXCL-8) is part of the CXC chemokine family, which enhances EC survival, proliferation, ECM production, and tube formation [12, 31]. IL-6 is capable of increasing endothelial permeability and migration, stimulating endothelial cell proliferation, and inducing tube formation in vitro and in vivo [31, 60, 61]. GROa/b/g (also known as CXCL-1/-2/-3, respectively) belong to the IL-8 angiogenic cytokine family. These factors can activate leukocyte migration, enhance endothelial cell chemotaxis, regulate inflammation and angiogenesis, mediate monocyte cell cycle arrest, and activate neutrophil cell migration [62]. CXCL5 is also part of the CXC chemokine family and controls angiogenic properties by enhancing microvascular endothelial cell migration and tube formation [31]. Monocyte chemotactic protein-1 (MCP-1) (also called chemokine (C-C motif) ligand 2, CCL2) upregulates FGF, PDGF, and VEGF expression; stimulates EC proliferation and migration; and induces MMP-1 secretion by ECs to degrade the ECM [63]. In addition to these five proangiogenic factors, the expression of three traditional proangiogenic factors in the sub-cultured placental tissue was also increased. Angiogenin, VEGF-A, and bFGF (FGF-2) are important players in endothelial cell-mediated angiogenesis, from degrading the basement membrane and activating angiogenic signaling transduction to promoting endothelial cell biological activities [47, 64]. μPAR induces endothelial cell proliferation and invasion in the early period of angiogenesis [31]. The process of primary hPDMSC isolation is complex and time-consuming; however, the process of obtaining the sub-cultured placental tissue-derived CM is easier, and it is easier to standardize production in batches. Therefore, sub-cultured placental tissue-derived CM has wider applications in vascular regeneration of tissue engineering and clinical angiogenesis therapy. Strong experimental and theoretical support for these applications was provided by our research.

While our study findings are novel and exciting, we recognize some limitations. Our study suggested that CM derived from placental cells or sub-cultured placental tissue affects HUVEC angiogenesis in vitro, but in vivo functional and molecular studies are needed for confirmation.

## Conclusions

In summary, CM from primary placental cells or sub-cultured full-term placental tissue contained proangiogenic factors and promoted HUVEC angiogenesis in vitro. Because of its cell-free, low immunogenicity, nontumorigenic nature, the simple preparation process and easily standardized mass production, sub-cultured placental tissue-derived CM has broad application prospects in tissue engineering and clinical angiogenesis therapy.

## Data Availability

All the datasets used and/or analyzed during this study are available from the corresponding authors on reasonable request.

## Abbreviations

CTBs: Cytotrophoblasts
hPDMSCs: Human placenta-derived mesenchymal stem cells
CM: Conditioned medium
HUVECs: Human umbilical vein endothelial cells
CXCL: Chemokine (C-X-C motif) ligand
GRO: Growth-regulated oncogene
IL: Interleukin
MCP: Monocyte chemoattractant protein
evCTBs: Extravillous cytotrophoblasts
DMEM: Dulbecco’s modified Eagle’s medium
FBS: Fetal bovine serum
PBS: Phosphate-buffered saline
PE: Phycoerythrin
FITC: Fluorescein isothiocyanate
rh-VEGF: Recombinant human vascular endothelial growth factor
rh-bFGF: Basic fibroblast growth factor
vWF: von Willebrand factor
CK: Cytokeratin
DAPI: 4′, 6-Diamidino-2-phenylindole
early-CTBs: Cytotrophoblast from early pregnancy placenta
early-hPDMSCs: Human mesenchymal stem cells from early pregnancy placenta
middle-hPDMSCs: Human mesenchymal stem cells from middle pregnancy placenta
term-hPDMSCs: Human mesenchymal stem cells from full-term placenta
IOD: Integrated optical density
bFGF: Basic fibroblast growth factor
TPO: Thrombopoietin
MMP: Matrix metalloproteinase
uPAR: Urokinase-type plasminogen activator receptor
PLGF: Placental growth factor
CCL: C-C motif chemokine ligand
ANGPT: Angiopoietin
GM-CSF: Granulocyte-macrophage colony-stimulating factor
